# Interpretation of the DOME Recommendations for Machine Learning in Proteomics and Metabolomics

**DOI:** 10.1021/acs.jproteome.1c00900

**Published:** 2022-02-04

**Authors:** Magnus Palmblad, Sebastian Böcker, Sven Degroeve, Oliver Kohlbacher, Lukas Käll, William Stafford Noble, Mathias Wilhelm

**Affiliations:** † Center for Proteomics and Metabolomics, Leiden University Medical Center, 2300 RC, Leiden, The Netherlands; ‡ Faculty of Mathematics and Computer Science, Friedrich Schiller University, 07743 Jena, Germany; § VIB-UGent Center for Medical Biotechnology, VIB, Ghent, Belgium and Department of Biomolecular Medicine, Ghent University, 9052 Ghent, Belgium; ∥ Eberhard Karls Universität Tübingen, WSI/ZBIT, 72076 Tübingen, Germany; ⊥ Science for Life Laboratory, School of Engineering Sciences in Chemistry, Biotechnology and Health, Royal Institute of Technology (KTH), 171 21 Solna, Sweden; # Department of Genome Sciences and the Paul G. Allen School of Computer Science and Engineering, University of Washington, Seattle, Washington 98195-5065, United States; ▽ Computational Mass Spectrometry, Technical University of Munich (TUM), 85354 Freising, Germany

## Abstract

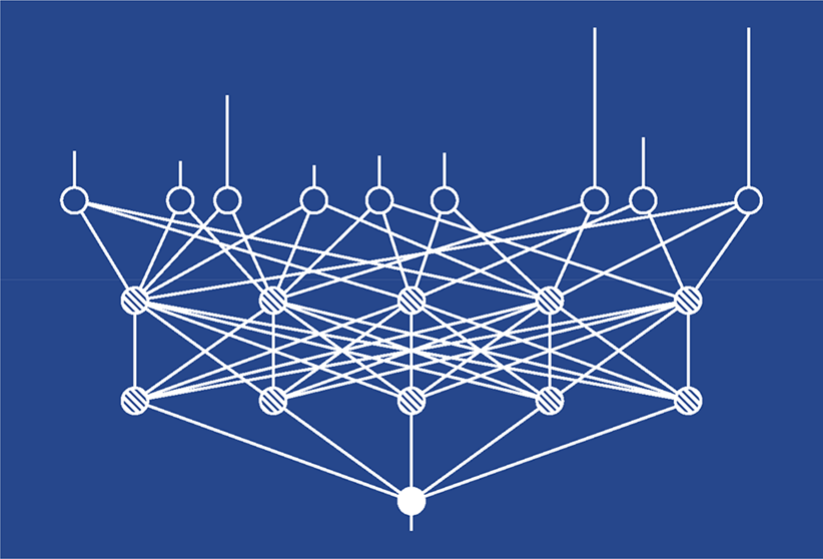

Machine learning is increasingly applied in proteomics and metabolomics to predict molecular structure, function, and physicochemical properties, including behavior in chromatography, ion mobility, and tandem mass spectrometry. These must be described in sufficient detail to apply or evaluate the performance of trained models. Here we look at and interpret the recently published and general DOME (Data, Optimization, Model, Evaluation) recommendations for conducting and reporting on machine learning in the specific context of proteomics and metabolomics.

The recently published DOME (Data, Optimization, Model, Evaluation) recommendations^1,2^ for reporting supervised machine learning (ML) research in biology aim to guide journal editors, reviewers, authors, and readers in understanding and comparing supervised ML methods and results in the biological sciences. The recommendations are designed to be general and therefore applicable to supervised ML in any biological discipline. This means that specific interpretations are needed to provide specific recommendations, examples, and guidance in fields where ML is seeing rapidly increased application. Proteomics and metabolomics are two such fields (Figure 1). Here we briefly summarize our interpretation of the DOME recommendations for the use and reporting of ML in proteomics and metabolomics with a few specific examples. Because they require different considerations, we will not cover clinical applications of ML. These are instead being addressed by other reporting standards and checklists such as MINIMAR,^3^ CONSORT-AI,^4^ SPIRIT-AI,^5^ and TRIPOD-ML.^6^

**Figure 1:**
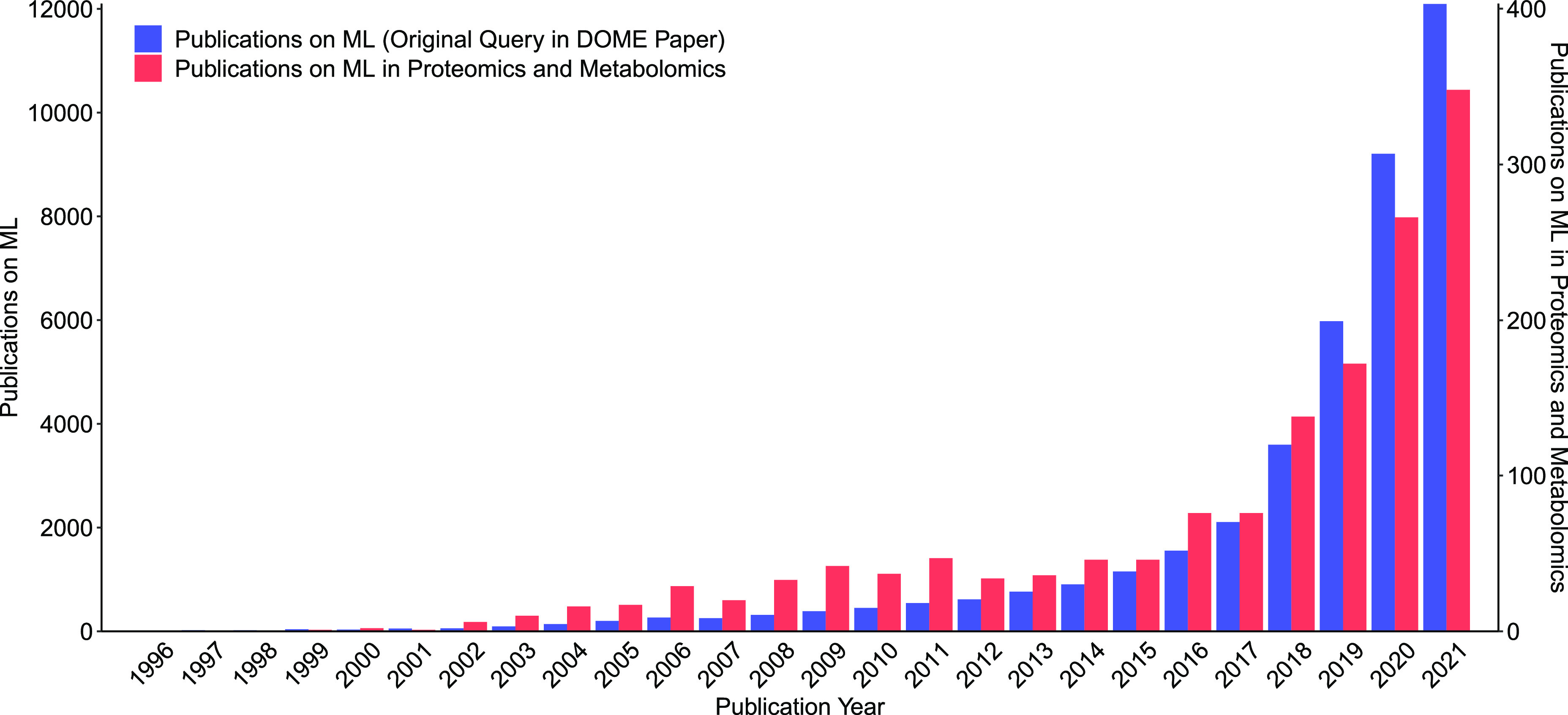
Number of publications on machine learning in proteomics or metabolomics has increased rapidly in the last 5 years, as revealed by a Web of Science literature search. The results also suggest an early ML hype in these domains around 2009–2011 and a shallow but noticeable “trough of disillusionment” from 2012 to 2013. The DOME query was the same as in the original paper [TS = “machine learning” AND ALL = (“biolog*” OR “medicine” OR “genom*” OR “prote*” OR “cell*” OR “post translational” OR “metabolic” OR “clinical”)]. The modified proteomics or metabolomics query was TS = “machine learning” AND ALL = (“biolog*” OR “medicine” OR “genom*” OR “prote*” OR “cell*” OR “post translational” OR “metabolic” OR “clinical”) AND ALL = (“proteome” OR “proteomics” OR “metabolome” OR “metabolomics” OR “metabonome” OR “metabonomics”). The searches were done on 2022-01-22.

ML efforts in proteomics and metabolomics share many challenges and considerations because the data are derived from similar methods of chromatography, ion mobility, and mass spectrometry. Although no meta-analysis of ML method descriptions in either domain has been published, there is no reason to assume the results would be much better than those in other domains. It was recently reported for metagenomics, for example, that only 12% of published papers use a proper test set for area under the receiver operating curve (AUC) reporting.^7^

In interpreting the DOME recommendations, we follow the same structure as that in the DOME publication, with acronymized broad topics Data, Optimization, Model, and Evaluation, including general things to “be on the lookout for” (or “BOLOs” for those familiar with law enforcement jargon) and specific recommendations for the application and description of ML in proteomics or metabolomics (Table 1).

**Table 1:** Specific Recommendations under the Broad Topics and BOLOs as in the DOME Recommendations^1^

broad topic	be on the lookout for	specific recommendations
**Data**	Data size and quality	Training data sufficiently represents the complexity of the modeled molecular class (e.g., tryptic peptides, lipids, all metabolites).
		Be clear if data used for training and testing are acquired on similar instruments (e.g., with the same mass analyzer) using similar settings (e.g., collision energy) or on a range of instruments or conditions.
		Beware of chimeric spectra and their possibly contaminating effects.
	Appropriate partitioning, dependence between train and test data	Training and test data should be disjoint on not only the spectrum level but also the molecular structure (e.g., peptide) level. Stereoisomers fragment highly similarly, and hence stereoisomers must not be present in the training and test sets to avoid biased statistics. Structural similarity or homology between training and test data should be kept to a minimum or should be controlled to mimic realistic test conditions.^8^
	No access to data	Training and test data are available in a public repository^9^ (e.g., the ProteomeXchange consortium^10^).
		If filtering or partitioning spectra in the same data sets, provide lists of Universal Spectrum Identifiers^11^ defining data used to train and test the model when available.^a^
	Other	Beware of redundancy in training or test data (e.g., multiple spectra of the same or similar molecular structures).
		Beware of false-positives and -negatives in training data and possible bias when selecting strict thresholds for compound identification.
		Beware of events affecting instrument performance over time, as those can artificially decrease or increase the apparent performance on an independent test set (e.g., instrument maintenance and calibration events).
**Optimization**	Overfitting, underfitting, and illegal parameter tuning	Compare with experimental variability. Is the claimed performance better than the expected experimental variability (e.g., in peak intensities or retention times)?
		Report any hyperparameter tuning (e.g., of deep neural network architectures).
	Imprecise parameters and protocols given	Define the optimization target (e.g., spectrum-, peptide-, or protein-level statistics).
		Provide the metric for comparing chromatograms or spectra (e.g., spectral angle, cosine score, or dot product) and a detailed description on how to apply it (e.g., if specific peaks for cosine score calculation were discarded, tolerances used for matching peaks, or strategies to resolve ambiguities).
**Model**	Unclear if black box (opaque) or interpretable (transparent) model	If the model is interpretable, describe how the trained model can be interpreted and what can be learned from it.
	No access to resulting source code and trained models	Specify which model, software, and version were used.
		Make documented source code publicly available.
	Execution time is impractical	Execution time for the training or application of a model should not be a bottleneck in its intended pipeline. As a rule of thumb, applying the model should not take longer than data acquisition. Execution time is even more critical in real-time applications such as continuous retention time alignment.
**Evaluation**	Performance measures inadequate	Motivate the use of performance measures, especially if reporting a single number. Report the Matthews correlation coefficient (MCC), not F1 scores or AUCs, for binary classifiers trained on classes of different sizes.^12^ The confusion matrix, from which other metrics can be calculated, can always be included.
	No comparisons to baselines or other methods	Compare performance with simpler baseline methods (e.g., linear regression predicting ion mobility using only mass or retention times using only amino acid composition).
		Include tests measuring performance of the algorithm in a practical user situation. For example, when predicting retention time, what increase in the number of identified or quantified compounds or peptides does the prediction imply?
		Evaluate model on independent test data acquired on a different instrument in a different lab.
		Do not include peaks corresponding to the precursor ion when comparing tandem mass spectra.
	Highly variable performance	Compare models on the same data. Make sure that the metric used for comparison is the same and was applied the same (e.g., do not compare cosine scores that were calculated based on different sets of ions). If a (community-developed) benchmark data set is available, then use it. If cross-validation is employed, then report the random splits (e.g., USIs) so that others can reproduce your work. (Communities are encouraged to develop benchmarking data sets for ML).
		Explicitly state model limitations (e.g., the type and conditions of chromatography for retention time prediction or the ionization mode, fragmentation, and mass analyzer for simulating tandem mass spectra).

a For training and test data sets containing millions of identified spectra, such Universal Spectrum Identifier (USI) lists will be very long. However, we expect that they would be primarily generated and read by machines, and even if they are long, USI lists take considerably less space than the mass spectra themselves. Furthermore, lists of USIs with many identifiers from the same data sets can be compressed by at least a factor of 5. More than 1 billion identifiers are already available in the ProteomeXchange repositories.

A few of the recommendations may be elaborated upon. For example, how do we know if the complexity of the modeled molecular class is sufficiently represented? For small molecules, this can be checked by comparing the coverage of compound classes according to ClassyFire^13^ or ChEBI^14^ with that of the intended application of the model. For peptides, a typical BOLO would be a model claimed to be applicable to all peptides trained exclusively on tryptic peptides. What constitutes a sufficient representation generally depends on both the model and its intended application.

How does one evaluate the presence of false-positives in the training data, for example, falsely identified tandem mass spectra or incorrectly assigned peaks? On one hand, such erroneously assigned spectra may confuse the model and should therefore be minimized. On the other hand, we should avoid overly stringent criteria that exclude compounds that intrinsically produce low-scoring compound–spectrum matches through poor or unusual fragmentation penalized in the initial scoring of the match. Ideally, practitioners should both provide a good estimate of the false-positive rate in the training data and show that the expected number of false-positives or mislabeled test data is not detrimental to the model.

As a final comment and following the recommendation to compare complex models with simple ones, we suggest that if a simple model reaches almost the same performance as a complex one, then one should always prefer the simple model, as it almost certainly has better generalizability (Occam’s razor).

Echoing suggestions by Walsh et al.^15^ and Jones^16^ and the efforts of the ELIXIR Machine Learning Focus Group^17^ and the AIMe registry,^18^ we believe the domain-specific interpretation of these guidelines will be helpful to authors, reviewers, and editors in preparing and evaluating manuscripts describing work involving ML in proteomics and metabolomics in this and other journals. These recommendations should not be interpreted as absolute requirements or minimum information about an ML application in these domains but rather as a helpful first checklist. As ML is seeing rapidly expanding application in proteomics and metabolomics, best practices and reporting standards will have to be revisited in coming years. It is our hope that these recommendations will serve as a good starting point for such discussions.
